# Déformation palpébrale en S révélant un adénome pléomorphe

**DOI:** 10.11604/pamj.2014.17.114.3948

**Published:** 2014-02-17

**Authors:** Hakima Elouarradi, Rajae Daoudi

**Affiliations:** 1 Université Mohammed V Souissi, Service d'Ophtalmologie A de l'hôpital des Spécialités, Centre hospitalier universitaire, Rabat, Maroc

**Keywords:** Adénome pléomorphe, déformation en S, paupiére, Pleomorphic adenoma, s-shape deformation, eyelid

## Image en medicine

Il s'agit d'un homme de 47 ans, consultant pour l'apparition d'une masse palpébrale supérieure droite depuis 4 mois, sans aucun signe fonctionnel associé. Cliniquement, il existe une déformation en S de la paupière supérieure droite (A), associée à une hypertrophie de la glande lacrymale à la palpation. Une TDM orbitaire (B) est réalisée, suivie d'une exérèse complète de la tumeur et complétée par une analyse anatomo-pathologique objectivant un adénome pléomorphe. L'adénome pléomorphe est la plus fréquente des tumeurs bénignes de la glande lacrymale, représentant 40% des tumeurs épithéliales. Autrefois appelé tumeur mixte bénigne, il représente 3 à 5% des tumeurs de l'orbite et survient le plus souvent chez des patients d'âge moyen. Sa prise en charge suit un protocole dont l'objectif est d'éviter la récidive et la transformation maligne. Toute biopsie ou exérèse incomplète est contre-indiquée en cas de processus de la glande lacrymale vu le risque de récidive et surtout de transformation maligne.

**Figure 1:**
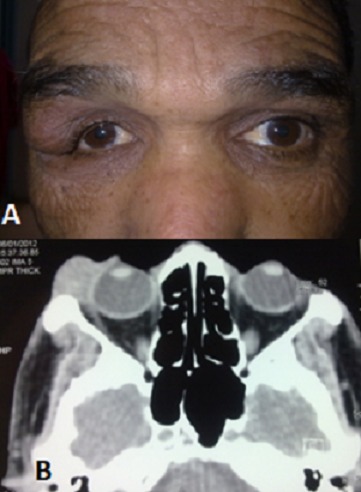
(A) Déformation en S de paupière droite et (B) Aspect scannographique objectivant un processus au dépend de la glande lacrymale

